# Fertility of Adults Born Very Preterm or With Very Low Birth Weight

**DOI:** 10.1001/jamanetworkopen.2025.1164

**Published:** 2025-03-19

**Authors:** Miranda Kit-Yi Wong, Nicole Tsalacopoulos, Peter Bartmann, Dieter Wolke

**Affiliations:** 1 Department of Psychology, University of Warwick, Coventry, United Kingdom; 2 Department of Pediatrics, University Hospital Würzburg, Würzburg, Germany; 3 School of Psychological Sciences, Monash University, Melbourne, Australia; 4 Department of Population Health Sciences, University of Leicester, Leicester, United Kingdom; 5 Department of Neonatology and Paediatric Intensive Care, University Hospital Bonn, Germany; 6 Division of Health Sciences, Warwick Medical School, University of Warwick, Coventry, United Kingdom

## Abstract

**Importance:**

Contradictory differences regarding fertility among adults born very preterm (VP; <32 weeks’ gestation) or with very low birth weight (VLBW; <1500 g) have been reported at different ages. Longitudinal investigations of fertility beyond ages 20 to 29 years are lacking.

**Objective:**

To assess whether VP and VLBW are associated with lower fertility up to age 35 years.

**Design, Setting, and Participants:**

This prospective population-based cohort study was conducted in Bavaria, Germany, with participants born between January 1985 and March 1986 who required admission to a children’s hospital within the first 10 days after birth. Healthy infants born at term (ie, at least 37 weeks’ gestation) in the same hospital and children alive at 6 years were selected as controls. Follow-up occurred repeatedly up to 34 to 35 years. Data were analyzed from July to December 2024.

**Exposures:**

Gestational age and birth weight.

**Main Outcomes and Measures:**

Cumulative incidence of having the first alive child (ie, fertility) was calculated using Kaplan-Meier estimation and the difference between participants born VP or with VLBW and term-born participants was compared with log-rank tests. Univariable and hierarchical multivariable Cox proportional hazards regressions were performed to estimate hazard ratios (HRs) for fertility associated with VP and VLBW adjusting for individual factors. Estimations were compared between early (<30 years) and late (≥30 years) reproductive windows.

**Results:**

A total of 414 participants (212 born VP or with VLBW and 202 term-born) were included (mean [SD] age, 34.67 [0.53] years; 216 [52.2%] female). Cumulative incidence of having the first alive child was lower in participants born VP or with VLBW throughout the follow-up. The association of VP and VLBW with lower fertility was significant during the late (HR, 0.46; 95% CI, 0.31-0.68) but not early (HR, 0.73; 95% CI, 0.47-1.14) reproductive window; this association attenuated after adjusting for neonatal factors (sex and family socioeconomic status) and childhood neurosensory impairment at step 2 (HR, 0.59; 95% CI 0.40-0.88), childhood family factors (eg, parenting) at step 3 (HR, 0.61; 95% CI, 0.40-0.92), and there was no longer an association after adjusting for sociodemographic factors (eg, partnering) at step 4 (HR, 0.78; 95% CI, 0.50-1.20). Partnering (ie, being married or cohabitating with a partner) had the largest magnitude association with fertility across both the early (HR, 7.87; 95% CI, 3.44-18.00) and late (HR, 3.95; 95% CI, 2.47-6.31) reproductive windows.

**Conclusions and Relevance:**

In this cohort study of participants born VP or with VLBW and term-born adults, VP and VLBW were associated with overall lower fertility. A major reason was an association of less partnering with lower fertility, suggesting that partnering is key to reproductive success.

## Key Points

**Question:**

Is being born very preterm (VP) or with very low birth weight (VLBW) associated with lower fertility across the reproductive window?

**Findings:**

In this cohort study of 212 individuals born VP or with VLBW and 202 term-born adults, being born VP or with VLBW was associated with lower fertility during the late (≥30 y) but not early (<30 y) reproductive window. Neurosensory impairment and not finding a romantic partner were the factors with the largest magnitude associations with lower fertility.

**Meaning:**

These findings suggest that adults born VP or with VLBW may not have lower fertility until their late reproductive window.

## Teaser

This cohort study analyzes associations of being born very preterm or having very low birth weight with fertility later in life among adults up to age 35 years in Bavaria, Germany.

## Introduction

Fertility is defined as demonstrated fecundity (ie, biologic capacity for reproduction) and measured by live births.^1^ Population-linked registry studies report an association of very preterm (VP) birth (<32 weeks’ gestation) or very low birth weight (VLBW; <1500 g) with lower fertility.^2,3,4,5,6,7,8^ However, this has only been partially replicated in prospective cohort studies.^9,10,11,12,13,14^ For example, cohort studies found no significant differences in fertility between adults born VP or with VLBW and term-born adults in their twenties.^9,11,12,13^ Moreover, when stratified by age, reports of 2 Swedish registry studies^3,5^ and a meta-analysis^15^ showed that VP and VLBW are associated with lower fertility only at age 25 years or older.

Evolutionary theories may explain timing and overall success in reproduction. Life history (LH) theory^16,17,18,19^ is concerned with the potential trade-offs of early or late reproduction (ie, fast vs slow LH strategy) to maximize reproductive success. It suggests that early life adversity (eg, VP or VLBW) indicating a higher mortality risk^20^ may orient individuals toward a fast LH strategy (ie, to reproduce early to reduce the risk of not reproducing at all). Darwin’s theory of sexual selection^21,22,23^ conceptualizes reproductive success via the processes of intrasexual competition and intersexual selection. Individuals born VP or with VLBW more often than term-born individuals experience a range of impairments in childhood^24^ and lower education and wealth by adulthood.^25^ Individuals born VP or with VLBW may thus compete poorly compared with their healthy, same-sex, term-born peers for a sexual or long-term romantic partner to reproduce.^15^ As such, we may hypothesize that if individuals born VP or with VLBW find a partner, they may reproduce early according to LH theory, but during the full reproductive window they may be less fertile (ie, not having any children) due to less partnering according to sexual selection theory.

Apart from being born VP or with VLBW, other adverse childhood experiences may contribute to fertility across the reproductive window.^16,26^ While low family socioeconomic status (SES) is associated with earlier reproduction,^27,28^ positive childhood family factors (eg, greater maternal sensitivity or fewer parental marital conflicts) may slow individual LH strategies.^29,30,31,32,33,34^ For partner selection, education and financial prospects are important cues to assess mate qualities.^35,36,37,38^ Higher education and income are associated with increased fertility in men^39,40,41^ but with delayed parenthood and fewer children in women.^39,42,43,44,45^

The present birth cohort study investigated the fertility of adults born VP or with VLBW vs adults born at term across the reproductive window until 34 to 35 years. We hypothesized that (1) VP and VLBW are associated with lower fertility during the overall reproductive window, (2) individuals born VP or with VLBW reproduce more or the same in their twenties but reproduce less during the late reproductive window, and (3) childhood and adulthood factors beyond VP and VLBW are associated with fertility.

## Methods

### Study Design, Setting, and Participants

**Figure 1.**
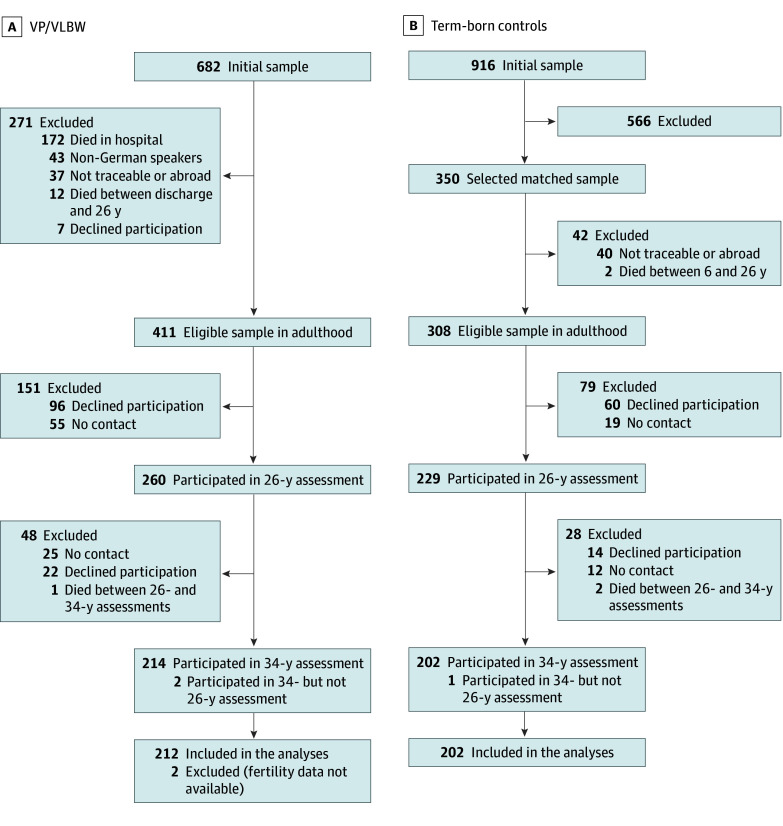
Participant Flowchart Flowchart describes eligible sample and those lost to follow-up among participants born very preterm or with very low birth weight (VP/VLBW) and term-born participants at 34-year assessment.

This cohort study was approved by the University of Munich Children’s Hospital and the University Hospital Bonn ethical review boards and followed the Strengthening the Reporting of Observational Studies in Epidemiology (STROBE) reporting guideline.^46^ Data were collected as part of the prospective Bavarian Longitudinal Study, a geographically defined whole-population cohort study of neonatal at-risk children born between January 1985 and March 1986 in Southern Bavaria, Germany, and who required admission to a children’s hospital within the first 10 days after birth (7505 individuals).^47,48^ Of those admitted, 682 infants were born VP or with VLBW and 411 were alive and eligible for follow-up in adulthood. Healthy infants born at term (ie, at least 37 weeks’ gestation) in the same obstetric hospitals were recruited as controls (916 individuals) and 350 children who were alive at 6 years were randomly selected as term controls. Of these, 308 were eligible for follow-up in adulthood (Figure 1). Informed written consent was obtained from parents at birth and from participants for the assessments in adulthood.

### Main Outcomes

Fertility was assessed repeatedly until 34 to 35 years with a standard life course interview adapted from established instruments.^49,50,51^ Fertility was defined as having the first alive biological child, and age was calculated as the difference between the birth dates of the participants and their first child.

### Study Variables

Study variables were collected at birth; 5, 20, and 56 months; and 6, 8, and 26 years. Data on gestational age (completed weeks), birth weight (grams), and sex were retrieved from birth records. Family SES was assessed at birth and computed as a weighted composite score of parental highest education and occupation, categorized as low, middle, and high.^52^ Neurosensory impairment (NSI) was diagnosed at 6 or 8 years if the child had any of the following: severe cerebral palsy (grade 3 or 4),^53^ hearing loss (uncorrected), blindness, or intelligence quotient (Kaufman Assessment Battery for Children mental processing composite score)^54^ less than 2 SD below the mean.^52^

Childhood family variables included sibling status, categorized as no sibling, having a sibling since infancy (at birth, 5 months, or 20 months), or having a sibling since childhood (at 56 months, 6 years, or 8 years). The Cognitively Stimulating Parenting Index^55,56^ was created based on items of the Home Observation for Measurement of the Environment Inventory^57^ at 6 years and refers to parental teaching efforts, the provision of teaching objects, and the quality of literacy and leisure habits. Mother-child interaction was observed at age 6 years and rated using an established standardized coding system, the Assessment of Mother-Child-Interaction with an Etch-a-Sketch.^58^ Rating scales consist of 3 subscales for the mother (verbal control, nonverbal control, and criticism; all reverse-coded) and 1 subscale for mother-child joint behavior (harmony), which were combined into an index scale of maternal sensitivity.^55,59,60^ The partner relationship quality of the participants’ parents was assessed at ages 6 and 8 years with the overall score of the Dyadic Adjustment Scale,^61^ which measures dyadic satisfaction, dyadic cohesion, dyadic consensus, and affectional expression. Low levels of cognitively stimulating parenting, maternal sensitivity, and parental partner relationship quality were defined as a score below the 25th percentile. For details of measurement description, please see the eMethods in Supplement 1.

Participants’ sociodemographic variables were collected at 26 years. Educational level was classified according to the International Standard Classification of Education (ISCED)^62^ as low (ISCED levels 0-2), medium (ISCED levels 3-5), and high (ISCED levels 6-8). Relative poverty was defined as net income for a 1-person household being 60% less than the average monthly household income of the total German population (<€980 in 2011). Partnership status was categorized as no partner vs being married or cohabiting; it was collected again at 34 years but was not used for modeling.

### Other Variables

At 26 and 34 years, participants were asked if they had ever experienced sexual intercourse and engaged in a serious romantic relationship, and if so, their age (years) at first sexual intercourse and first serious romantic relationship. History of miscarriage, stillbirth, and abortion was obtained at 34 years.

### Statistical Analysis

All participants with relevant fertility data were included in the analysis. Missing data (12.1%) were imputed using multiple imputation by chained equations (mice).^63^ We computed descriptive statistics for participant characteristics and calculated cumulative incidence of having the first alive child stratified by birth group (VP or VLBW vs term-born) using Kaplan-Meier estimation and compared the group difference with a log-rank test. To assess the association of VP, VLBW, and individual factors with fertility, we performed univariable and hierarchical multivariable Cox proportional hazards regressions to estimate hazard ratios (HRs) and 95% CIs, with birth group entered at step 1, neonatal factors (sex and family SES) and childhood NSI at step 2, childhood family factors (sibling status, cognitively stimulating parenting, maternal sensitivity, and parental partner relationship quality) at step 3, and sociodemographic factors (educational level, relative poverty, and partnership status) at step 4. A likelihood ratio test was used to assess if each step improved the ability of the model to explain variability in fertility. To assess the extent to which individual factors explained the association of VP and VLBW with fertility, we calculated the percentage of excess risk mediated (PERM)^64^ as:

PERM = (HR_confounder adjusted_ − HR_confounder and mediator adjusted_) / (HR_confounder adjusted_ − 1) × 100.

A landmark approach^65,66^ was applied to determine the association change before and after the landmark time. The average age of first-time motherhood in Germany is between 29 and 30 years,^67^ so the landmark time used was 30 years to differentiate the early (<30y) vs late (≥30y) reproductive window. Follow-up for the overall reproductive window started at 18 years and continued until having the first child or the 34-year assessment (whichever was earlier); follow-up for the early reproductive window continued until having the first child or age 29 years (whichever was earlier) including all participants. For the investigation of late reproductive window, only including participants who remained childless at age 29 years, follow-up started at 30 years and continued until having the first child or the 34-year assessment (whichever was earlier).

Two sensitivity analyses were conducted: (1) using inverse probability weighting to adjust for potential selective dropout (eAppendix in Supplement 1) and (2) a subgroup analysis among participants who were partnered (ie, married or cohabiting) at age 34 years.

All analyses were performed in R version 4.3.2 (R Project for Statistical Computing) from July to December 2024. Statistical significance was set as a 2-tailed *P* < .05.

## Results

### Participant Characteristics and Dropout Analysis

There were 414 participants (212 born VP or with VLBW and 202 term-born) included in the analyses (Figure 1). The mean (SD) age at last assessment was 34.67 (0.53) years (range, 33.78-36.58 years) and 216 participants (52.2%) were female. Table 1 shows the participant characteristics stratified by birth group. Compared with term-born participants, fewer participants born VP or with VLBW were born into families of high SES, were without a childhood NSI, achieved a high level of education, and had partnering experiences (ie, experienced sexual intercourse, engaged in a serious romantic relationship, and were married or cohabiting) (eTable 1 and eFigure 1 in Supplement 1). Eligible participants who were lost to follow-up by the 34-year assessment were compared with participants included in the analyses on neonatal variables and childhood NSI. Loss to follow-up was associated with low family SES in adults born VP or with VLBW and with male sex and low family SES in term-born adults (eTable 2 in Supplement 1).

**Table 1.** Characteristics of Participants Included in the Analyses (Stratified by Birth Group)

Characteristic	Participants, No./Total No. (%)		*P* value^a^	Missing, No. (%) (N = 414)
	VP/VLBW (n = 212)	Term-born (n = 202)		
Neonatal characteristics				
Gestational age, mean (SD), wk	30.53 (2.06)	39.64 (1.19)	<.001	0
Birth weight, mean (SD), g	1317.95 (311.43)	3360.69 (460.07)	<.001	0
Sex				
Female	103/212 (48.6)	113/202 (55.9)	.13	0
Male	109/212 (51.4)	89/202 (44.1)		
Family SES				
High	49/212 (23.1)	69/202 (34.2)	.04	0
Middle	103/212 (48.6)	87/202 (43.1)		
Low	60/212 (28.3)	46/202 (22.8)		
Childhood and family characteristics				
Presence of childhood NSI	43/209 (20.6)	1/202 (0.5)	<.001	3 (0.7)
Sibling status				
No sibling	40/212 (18.9)	22/202 (10.9)	<.001	0
Had sibling at infancy	121/212 (57.1)	91/202 (45)		
Had sibling in childhood	51/212 (24.1)	89/202 (44.1)		
Low level of cognitively stimulating parenting	41/182 (22.5)	24/202 (11.9)	.01	30 (7.2)
Low level of maternal sensitivity	56/176 (31.8)	38/202 (18.8)	.004	36 (8.7)
Low level of parental partner relationship quality	47/193 (24.4)	35/189 (18.5)	.17	NA^b^
Adulthood characteristics				
Age at assessment, mean (SD), y	34.66 (0.54)	34.68 (0.53)	.75	0
Highest level of education achieved^c^				
High	36/210 (17.1)	74/201 (36.8)	<.001	3 (0.7)
Medium	138/210 (65.7)	102/201 (50.7)		
Low	36/210 (17.1)	25/201 (12.4)		
Relative poverty	72/204 (35.3)	55/195 (28.2)	.13	15 (3.6)
Sexual intercourse				
26 y	156/209 (74.6)	200/201 (99.5)	<.001	4 (1.0)
34 y	169/206 (82.0)	202/202 (100.0)	<.001	6 (1.4)
Age at first sexual intercourse, mean (SD), y	18.46 (3.30)	16.73 (1.85)	<.001	NA^d^
Serious romantic relationship				
26 y	158/210 (75.2)	194/201 (96.5)	<.001	3 (0.7)
34 y	168/210 (80.0)	199/202 (98.5)	<.001	2 (0.5)
Age at first serious romantic relationship, mean (SD), y	18.46 (3.83)	16.98 (2.44)	<.001	NA^e^
Married or cohabitating				
26 y	107/210 (51.0)	132/201 (65.7)	.002	3 (0.7)
34 y	123/212 (58.0)	173/202 (85.6)	<.001	0
Fertility characteristics				
First alive child				
Had first alive child by 34-y assessment	76/212 (35.8)	114/202 (56.4)	<.001	0
Had first alive child <30 y	36/76 (47.4)	46/114 (40.4)	.34	0
Age at having the first alive child, mean (SD), y	29.82 (3.64)	30.46 (3.28)	.21	0
Miscarriage or stillbirth in the past	13/98 (13.3)	12/113 (10.6)	.55	NA^f^
Abortion in the past	5/98 (5.1)	3/113 (2.7)	.35	NA^f^

Abbreviations: NA, not applicable; NSI, neurosensory impairment; SES, socioeconomic status; VP/VLBW, very preterm or very low birth weight.

^a^ Independent sample *t* test for continuous variables and χ^2^ test for categorical variables were performed.

^b^ Participants whose parents had no partner at the time of assessment.

^c^ Educational level was classified according to the International Standard Classification of Education (ISCED) as low (ISCED levels 0-2), medium (ISCED levels 3-5), and high (ISCED levels 6-8).

^d^ Participants who had never experienced sexual intercourse at the time of assessment.

^e^ Participants who had never engaged in a serious romantic relationship at the time of assessment.

^f^ Participants who were never pregnant or whose partner was never pregnant at the time of assessment.

### First Reproductive Event

**Figure 2.**
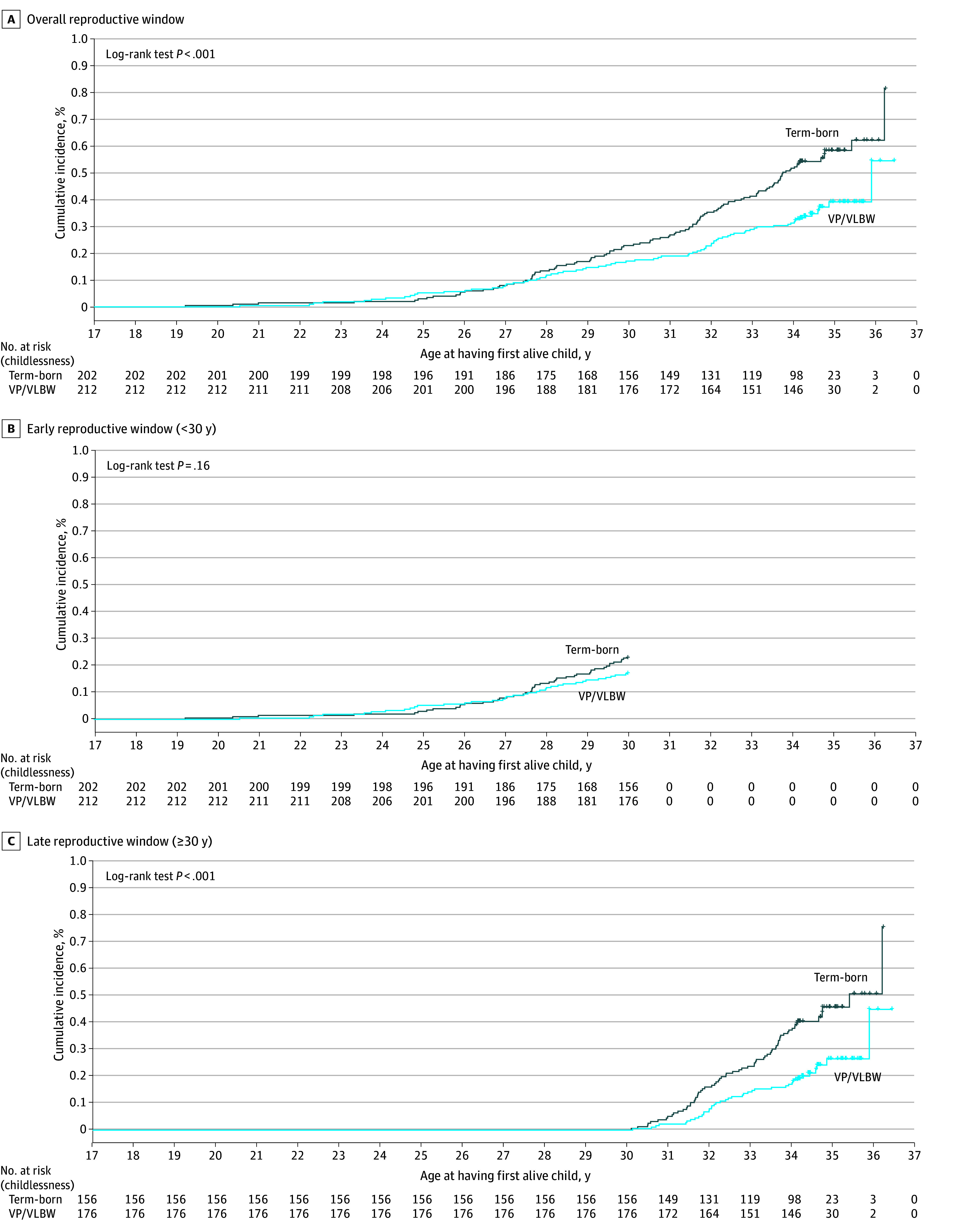
Cumulative Incidence of Having the First Alive Child The figure shows cumulative incidence of having the first alive child in participants born very preterm (VP) or with very low birth weight (VLBW) and term-born participants during the overall (A), early (B), and late (C) reproductive windows.

Compared with term-born participants, fewer participants born VP or with VLBW had at least 1 alive child (76 participants [35.8%] vs 114 participants [56.4%]; *P* < .001) (Table 1). Among those who were parents, participants born VP or with VLBW had their first alive child slightly earlier than term-born participants (mean [SD] age, 29.82 [3.64] years vs 30.46 [3.28] years; *P* = .21), and a slightly higher proportion of participants born VP or with VLBW had their first alive child before 30 years (36 of 76 participants [47.4%] vs 46 of 114 participants [40.4%]; *P* = .34), although these findings were not significant. Figure 2 illustrates the cumulative incidence of having the first alive child stratified by birth group, with log-rank tests revealing significant variations during the overall (*P* < .001) and late reproductive window (*P* < .001) but not the early reproductive window (*P* = .16).

### Association of VP, VLBW, and Individual Factors With Fertility

**Figure 3.**
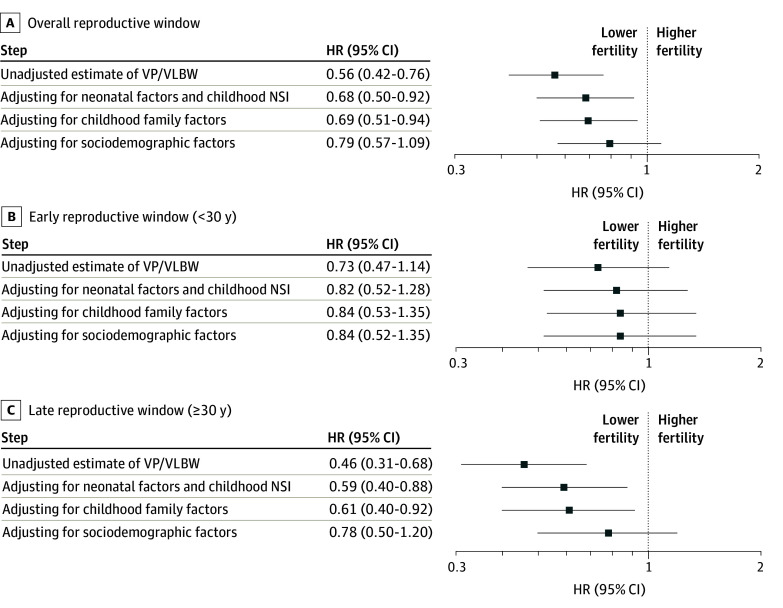
Association of Fertility With Being Born Very Preterm or With Very Low Birth Weight (VP/VLBW) The figure shows hazard ratios (HRs) and 95% CIs at each step of the hierarchical multivariable Cox proportional hazards regression models during the overall (A), early (B), and late (C) reproductive windows. NSI indicates neurosensory impairment.

Figure 3 shows the association of VP and VLBW with fertility at each step estimated by the hierarchical multivariable Cox proportional hazards regressions. Initially, at step 1, VP and VLBW were associated with lower fertility during the overall (HR, 0.56; 95% CI, 0.42-0.76) and late (HR, 0.46; 95% CI, 0.31-0.68) reproductive window but not early reproductive window (HR, 0.73; 95% CI, 0.47-1.14). This pattern remained after adjusting for neonatal factors and childhood NSI at step 2 (overall: HR, 0.68; 95% CI, 0.50-0.92; late: HR, 0.59; 95% CI 0.40-0.88) and childhood family factors at step 3 (overall: HR, 0.69; 95% CI, 0.51-0.94; late: HR, 0.61; 95% CI, 0.40-0.92); however, the increasing adjusted HRs indicated that the adjusting factors attenuated this association. Finally, at step 4, after adjusting for sociodemographic factors, there was no longer an association of VP and VLBW with lower fertility during the overall (HR, 0.79; 95% CI, 0.57-1.09) and late (HR, 0.78; 95% CI, 0.50-1.20) reproductive window.

Likelihood ratio tests showed significantly increased variance in explaining fertility at step 4 (eTable 3 in Supplement 1). Thus, we investigated which individual factors beyond VP and VLBW were associated with fertility and compared the early and late reproductive windows.

Table 2 presents the results of the univariable and multivariable (final model [step 4]) analyses examining the association of VP, VLBW, and individual factors with fertility during the early and late reproductive windows (see eTable 4 in Supplement 1 for the overall reproductive window). The multivariable analysis found that higher fertility was associated with being married or cohabiting during both the early (HR, 7.87; 95% CI, 3.44-18.00) and late (HR, 3.95; 95% CI, 2.47-6.31) reproductive windows; male sex and low family SES were only significant during the early (male sex: HR, 0.40, 95% CI, 0.24-0.65; low family SES: HR, 2.24; 95% CI, 1.07-4.66) but not late (male sex: HR, 0.96; 95% CI, 0.65-1.43; low family SES: HR, 1.13; 95% CI, 0.66-1.93) reproductive window, while presence of childhood NSI was only significant during the late (HR, 0.08; 95% CI, 0.01-0.57) but not early (HR, 0.50; 95% CI, 0.15-1.64) reproductive window. The association of education with fertility was only significant during the early reproductive window and was nonlinear; higher fertility was associated with medium level of education (HR, 2.00; 95% CI, 1.09-3.67). No childhood family factors were associated with fertility. The association of relative poverty with fertility during the late reproductive window was significant in univariable analysis (HR, 0.55; 95% CI, 0.35-0.87) but not in multivariable analysis (HR, 0.61; 95% CI, 0.35-1.04) despite similar effect size. The overall attenuation in the association of VP and VLBW with lower fertility during the late reproductive window by the presence of childhood NSI and being married or cohabiting was substantial with a PERM of 54.2% (eTable 5 in Supplement 1).

**Table 2.** Univariable and Multivariable Analyses of Association of VP/VLBW and Individual Factors With Fertility During the Early (<30 y) and Late (≥30 y) Reproductive Windows

Factor	Univariable analysis^a^				Multivariable analysis			
	Early reproductive window		Late reproductive window		Early reproductive window		Late reproductive window	
	HR (95% CI)	*P* value	HR (95% CI)	*P* value	HR (95% CI)	*P* value	HR (95% CI)	*P* value
VP/VLBW	0.73 (0.47-1.14)	.17	0.46 (0.31-0.68)	<.001	0.84 (0.52-1.35)	.47	0.78 (0.50-1.20)	.26
Neonatal factors and childhood NSI								
Male sex	0.39 (0.24-0.63)	<.001	0.88 (0.60-1.29)	.51	0.40 (0.24-0.65)	<.001	0.96 (0.65-1.43)	.86
Family SES								
High	1 [Reference]	NA	1 [Reference]	NA	1 [Reference]	NA	1 [Reference]	NA
Middle	2.36 (1.24-4.48)	.009	0.89 (0.57-1.38)	.60	1.92 (0.98-3.78)	.06	1.07 (0.67-1.73)	.77
Low	3.01 (1.53-5.91)	.001	1.08 (0.65-1.77)	.78	2.24 (1.07-4.66)	.04	1.13 (0.66-1.93)	.67
Presence of childhood NSI	0.30 (0.09-0.94)	.04	0.06 (0.01-0.40)	.004	0.50 (0.15-1.64)	.25	0.08 (0.01-0.57)	.01
Childhood family factors								
Sibling status								
No sibling	1 [Reference]	NA	1 [Reference]	NA	1 [Reference]	NA	1 [Reference]	NA
Had sibling since infancy	0.73 (0.39-1.36)	.32	1.29 (0.69-2.40)	.43	0.67 (0.35-1.27)	.22	1.40 (0.72-2.69)	.32
Had sibling since childhood	1.07 (0.57-2.00)	.83	1.70 (0.89-3.24)	.11	0.88 (0.46-1.69)	.71	1.79 (0.91-3.52)	.09
Low level of cognitively stimulating parenting	1.24 (0.71-2.18)	.46	0.96 (0.57-1.62)	.88	1.05 (0.57-1.93)	.88	0.89 (0.51-1.58)	.70
Low level of maternal sensitivity	0.84 (0.49-1.44)	.53	1.01 (0.65-1.58)	.96	0.82 (0.46-1.47)	.51	1.30 (0.80-2.13)	.30
Low level of parental partner relationship quality	1.15 (0.68-1.95)	.60	0.96 (0.59-1.57)	.88	1.39 (0.81-2.37)	.23	1.09 (0.65-1.83)	.75
Sociodemographic factors								
Highest level of education achieved^b^								
High	1 [Reference]	NA	1 [Reference]	NA	1 [Reference]	NA	1 [Reference]	NA
Medium	1.81 (1.06-3.10)	.03	0.72 (0.47-1.09)	.12	2.00 (1.09-3.67)	.03	0.79 (0.49-1.27)	.32
Low	0.31 (0.09-1.05)	.06	0.54 (0.29-1.00)	.049	0.43 (0.12-1.53)	.20	1.35 (0.67-2.69)	.40
Relative poverty	0.79 (0.48-1.31)	.37	0.55 (0.35-0.87)	.01	1.40 (0.81-2.41)	.24	0.61 (0.35-1.04)	.07
Married or cohabiting	10.51 (4.58-24.15)	<.001	4.39 (2.78-6.92)	<.001	7.87 (3.44-18.00)	<.001	3.95 (2.47-6.31)	<.001

Abbreviations: HR, hazard ratio; NA, not applicable; NSI, neurosensory impairment; SES, socioeconomic status; VP/VLBW, very preterm or very low birth weight.

^a^ Participants with missing data were not included in the univariable analysis.

^b^ Educational level was classified according to the International Standard Classification of Education (ISCED) as low (ISCED levels 0-2), medium (ISCED levels 3-5), and high (ISCED levels 6-8).

### Sensitivity Analyses

The results were unchanged in sensitivity analysis using inverse probability weighting to adjust for potential selective dropout (eFigure 2 and eTable 6 in Supplement 1). Furthermore, just including those who were partnered, being born VP or VLBW was not differently associated with fertility compared with being term-born (eFigure 3 and eTable 7 in Supplement 1).

## Discussion

This prospective population-based cohort study examined fertility of adults born VP or with VLBW up to age 35 years and whether differences in fertility in comparison with term-born adults may be associated with individual functioning or social factors across childhood into adulthood. We found that VP and VLBW were associated with overall lower fertility, and this association emerged during the late but not early reproductive window. Adjusting for sociodemographic factors significantly narrowed the fertility gap between adults born VP or with VLBW and adults born at term. We identified partnering (ie, married or cohabiting) as the factor with the largest magnitude association with fertility across early and late reproductive windows. Furthermore, sex, family SES, and educational success were independently associated with fertility during the early reproductive window, while childhood NSI was associated with fertility during the late reproductive window.

Our results support the hypothesis that VP and VLBW are associated with overall lower fertility. Lower fertility in participants born VP or with VLBW did not become evident until their late reproductive window (≥30 years). This pattern reconciles previous results, which showed an unclear picture in early adulthood but lower fertility across adulthood overall in individuals born VP or with VLBW^3,5,9,11,12,13,14,15^; this appears to be a consequence of 2 different forces suggested by LH theory and sexual selection theory. As an adaptive strategy, LH theory suggests that individuals born VP or with VLBW, once partnered, may reproduce earlier to avoid not leaving any descendants at all. Indeed, an association of low birth weight with teenage motherhood (<20y)^33,68^ and individuals born VP or with VLBW having their first child earlier than the controls^6,69^ have been reported. Our results also indicate a slightly earlier age at having the first alive child and slightly more individuals born VP or with VLBW had their first alive child before 30 years, suggestive of an accelerated reproductive pattern.

Sexual selection theory proposes that individuals born VP or with VLBW may be outcompeted by their healthy, same-sex, term-born peers for a partner to reproduce. Even though individuals born VP or with VLBW may intend to reproduce earlier,^70^ this intention may not be actualized,^33^ if they do not find a cohabiting partner. Two recent meta-analyses^15,71^ reported that fewer individuals born VP or with VLBW ever experienced sexual intercourse or found a long-term partner by their twenties, a finding replicated here. The fertility gap was significantly reduced after adjusting for sociodemographic factors with partnering by far the largest-magnitude factor. Individuals born VP or with VLBW who were able to find a long-term partner were as fertile as term-born individuals even during their late reproductive window. Subgroup analysis among those who were partnered further indicated that, once partnered, being born VP or with VLBW was irrelevant to fertility.

What other factors contribute to fertility? Our results suggest that male sex, low family SES, and educational success may be of relevance during the early reproductive window, while childhood NSI may be relevant during the late reproductive window. Females tend to have children with males that are, on average, 2 to 3 years older,^72^ thus it is not surprising that males were less fertile during the early reproductive window; this sex difference disappeared during the late reproductive window. Consistent with previous studies,^27,28^ we found that low family SES was associated with increased fertility during the early reproductive window, contributing evidence to LH theory, which suggests that adverse childhood experiences encourage early reproduction.^27^ In addition, our result showed that a medium level of education was associated with increased fertility during the early reproductive window, aligning with previous results of nonlinear association of education with fertility.^39,40,41^ Higher education is a preferred mate quality,^35,36,37,38^ yet staying longer in education does delay parenthood.^39,42,43,44,45^ Fewer individuals born VP or with VLBW have higher education qualification^25^ and we found this was associated with earlier reproduction. Moreover, presence of NSI, which is more frequent in individuals born VP or with VLBW,^24^ is associated with fertility; this may make individuals born VP or with VLBW less attractive as a mate,^15,71^ which in turn lowers their fertility across the reproductive window. Indeed, presence of NSI and partnering appear to be the factors which mostly explained the association of VP and VLBW with lower fertility.

### Limitations

This study has limitations. There was selective loss to follow-up according to neonatal and social risks; this is found in all prospective studies.^72,73^ However, a repeated participation of more than 50% over 34 to 35 years is as high or higher than most cross-sectional studies achieve.^73^ Delayed parenthood in high-income countries^43,44^ may require longer follow-up beyond participants’ mid-thirties to evaluate the association of VP and VLBW with fertility. However, our data are among the best available cohort study data of individuals born VP or with VLBW in the world and we successfully replicated previous findings of lower fertility by participants’ mid-thirties. We did not collect data on additional confounders (eg, contraceptive use or fertility treatment), leaving potential for residual confounding. However, these may only be relevant if differently distributed between individuals born VP or with VLBW and term-born controls. The study region was limited to Bavaria, a federal state with a lower rate of single mothers; thus, cultural differences in reproduction may limit the generalizability of our results.

## Conclusions

This cohort study found that the fertility of individuals born VP or with VLBW may not have lower fertility until their late reproductive window (≥30 years). Our findings suggest that partnering was key to reproductive success. Those who attained a medium level of education and were without childhood NSI were more likely to find a partner and reproduce. To increase fertility among individuals born VP or with VLBW, we may consider new approaches such as specialized dating apps to facilitate partnering.

## Notes

Supplement 1. **eMethods.** Measurement Description of Cognitively Stimulating Parenting, Maternal Sensitivity, and Parental Partner Relationship Quality **eAppendix.** Inverse Probability Weighting **eTable 1.** Partnering Experiences of Full Sample for the 26- and 34-Year Assessments **eFigure 1.** Cumulative Incidence of First Partnering Experiences **eTable 2.** Characteristics of Eligible Participants for the 34-Year Assessment **eTable 3.** Likelihood Ratio Tests of the Hierarchical Multivariable Cox Proportional Hazards Regression Models **eTable 4.** Univariable and Multivariable Analyses of Association of VP/VLBW and Individual Factors With Fertility During the Overall Reproductive Window **eTable 5.** Percentage of Excess Risk Mediated by the Presence of Childhood NSI and Partnering in the Association of VP/VLBW With Lower Fertility During the Late Reproductive Window **eFigure 2.** Sensitivity Analysis of Association of Fertility with VP/VLBW Estimated Using Inverse Probability Weighting **eTable 6.** Sensitivity Analysis of Association of VP/VLBW and Individual Factors With Fertility Estimated Using Inverse Probability Weighting **eFigure 3.** Subgroup Analysis of Association of Fertility With VP/VLBW Among Participants Who Were Partnered at 34-Year Assessment **eTable 7.** Subgroup Analysis of Association of VP/VLBW and Individual Factors With Fertility Among Participants Who Were Partnered at 34-Year Assessment

Supplement 2.
Data Sharing Statement
