# Supplementary material for: Fertility of Adults Born Very Preterm or With Very Low Birth Weight
Source: JAMA Netw Open. 2025 Mar 19;8(3):e251164. doi: 10.1001/jamanetworkopen.2025.1164 (PMC11923700; doi:10.1001/jamanetworkopen.2025.1164)
Supplement: Supplement 1. — eMethods. Measurement Description of Cognitively Stimulating Parenting, Maternal Sensitivity, and Parental Partner Relationship Quality eAppendix. Inverse Probability Weighting eTable 1. Partnering Experiences of Full Sample for the 26- and 34-Year Assessments eFigure 1. Cumulative Incidence of First Partnering Experiences eTable 2. Characteristics of Eligible Participants for the 34-Year Assessment eTable 3. Likelihood Ratio Tests of the Hierarchical Multivariable Cox Proportional Hazards Regression Models eTable 4. Univariable and Multivariable Analyses of Association of VP/VLBW and Individual Factors With Fertility During the Overall Reproductive Window eTable 5. Percentage of Excess Risk Mediated by the Presence of Childhood NSI and Partnering in the Association of VP/VLBW With Lower Fertility During the Late Reproductive Window eFigure 2. Sensitivity Analysis of Association of Fertility with VP/VLBW Estimated Using Inverse Probability Weighting eTable 6. Sensitivity Analysis of Association of VP/VLBW and Individual Factors With Fertility Estimated Using Inverse Probability Weighting eFigure 3. Subgroup Analysis of Association of Fertility With VP/VLBW Among Participants Who Were Partnered at 34-Year Assessment eTable 7. Subgroup Analysis of Association of VP/VLBW and Individual Factors With Fertility Among Participants Who Were Partnered at 34-Year Assessment [file jamanetwopen-e251164-s001.pdf]

Supplemental Online Content

Wong, MKY, Tsalacopoulos N, Bartmann P, Wolke D. Fertility of adults born very preterm or with very low birth weight. *JAMA Netw. Open*. 2025;8(3):e251164.
doi:10.1001/jamanetworkopen.2025.1164

eMethods. Measurement Description of Cognitively Stimulating Parenting, Maternal Sensitivity, and Parental Partner Relationship Quality
eAppendix. Inverse Probability Weighting
eTable 1. Partnering Experiences of Full Sample for the 26- and 34-Year Assessments
eFigure 1. Cumulative Incidence of First Partnering Experiences
eTable 2. Characteristics of Eligible Participants for the 34-Year Assessment
eTable 3. Likelihood Ratio Tests of the Hierarchical Multivariable Cox Proportional Hazards Regression Models
eTable 4. Univariable and Multivariable Analyses of Association of VP/VLBW and Individual Factors With Fertility During the Overall Reproductive Window
eTable 5. Percentage of Excess Risk Mediated by the Presence of Childhood NSI and Partnering in the Association of VP/VLBW With Lower Fertility During the Late Reproductive Window
eFigure 2. Sensitivity Analysis of Association of Fertility with VP/VLBW Estimated Using Inverse Probability Weighting
eTable 6. Sensitivity Analysis of Association of VP/VLBW and Individual Factors With Fertility Estimated Using Inverse Probability Weighting
eFigure 3. Subgroup Analysis of Association of Fertility With VP/VLBW Among Participants Who Were Partnered at 34-Year Assessment
eTable 7. Subgroup Analysis of Association of VP/VLBW and Individual Factors With Fertility Among Participants Who Were Partnered at 34-Year Assessment

This supplemental material has been provided by the authors to give readers additional information about their work.

© 2025 Wong, MKY et al. *JAMA Network Open*.

eMethods. Measurement Description of Cognitively Stimulating Parenting, Maternal Sensitivity, and Dyadic Adjustment

Cognitively Stimulating Parenting

As part of the structured Mannheimer Parent Interview (MEI), a gold-standard interview in Germany, 31 questions of the Home Observation for Measurement of the Environment (HOME) Inventory (Caldwell & Bradley, 2004) were administered to parents. Response formats to these questions varied between dichotomous options (yes/no) and ordinal Likert-type scales. The original HOME items encompass three main categories that address (1) items that describe the availability and variety of experience that influence the level of cognitive stimulation in the home; (2) social items describing parent-child interactions; and (3) items describing the organisation of the physical environment.

Based on these HOME items administered at age 6 years an index scale of Cognitively Stimulating Parenting was created. The scale consists of 21 of the original HOME items, which asked the parent to self-report on the child's home literacy environment, the quality of learning materials provided, whether the parent was "trying or had tried to teach" the child a variety of concepts such as numbers and the alphabet, and the number of trips the parent had taken the child on. Accordingly, the crucial characteristics of the cognitively stimulating parenting scale are parental teaching efforts, the provision of teaching objects, and the quality of literacy and leisure habits (Bonthrone et al., 2021; Wolke et al., 2013).

Maternal Sensitivity

Participants were recorded during a standardised play situation using an Etch-a-Sketch, a toy that allows to produce pictures by means of two buttons, one that draws horizontal lines and one that draws vertical lines. The instruction was that the mother should use one button and the child the other button, thus both had to work together to copy the template. The use of different buttons by mother or child was counter-balanced across dyads and groups. If after 12 minutes there was no complete picture, the session was stopped. Individual sessions took place in quiet, specially equipped, and dedicated assessment rooms with appropriate lighting. Mother-child interaction was evaluated in vivo by experienced raters (psychologists) who were blind to group and family characteristics, with a standardised coding system, the "Assessment of Mother-Child-Interaction with the Etch-a-Sketch" (AMCIES) (Jaekel et al., 2012; Wolke et al., 1995). All mother-child interactions were video-recorded. Rating scales consisted of three subscales for the mother (verbal control, non-verbal control, and criticism, all reverse-coded) and one subscale for mother-child joint behaviour (harmony) (Jaekel et al., 2015; Wolke et al., 2013). These were *z*-standardised and combined into one index scale of Maternal Sensitivity (Jaekel et al., 2021). The AMCIES coding system has established high interrater reliabilities (Jaekel et al., 2012). For a subsample (*n*=565), the in-vivo rated scores

© 2025 Wong, MKY et al. *JAMA Network Open*.

were compared with video-rated scores of Maternal Sensitivity (Wolke et al., 2013) and showed excellent convergence (intraclass correlation coefficient = .76, $P < .001$).

Parental Partner Relationship Quality

The Dyadic Adjustment Scale (DAS; Spanier, 1976) is a 32-item instrument used for assessing dyadic adjustment, i.e. the quality of relationships in marriages and other similar dyads (i.e., cohabitating couples). The DAS conceptualises the quality of relationships into four subscales (using factor analysis), which are conceptually and empirically related to dyadic adjustment: dyadic satisfaction, dyadic cohesion, dyadic consensus, and affectional expression. The BLS added a further six items to the questionnaire. One item about parenting was added to the dyadic consensus subscale and five items to the dyadic satisfaction subscale. Summary scores are produced for each subscale as well as an overall DAS score.

References

Bonthrone, A. F., Chew, A., Kelly, C. J., Almedom, L., Simpson, J., Victor, S., Edwards, A. D., Rutherford, M. A., Nosarti, C., & Counsell, S. J. (2021). Cognitive function in toddlers with congenital heart disease: The impact of a stimulating home environment. *Infancy*, *26*(1), 184-199. https://doi.org/10.1111/infa.12376

Caldwell, B. M., & Bradley, R. H. (1979). *Home observation for measurement of the environment*. University of Arkansas at Little Rock Little Rock, AR.

Jaekel, J., Pluess, M., Belsky, J., & Wolke, D. (2015). Effects of maternal sensitivity on low birth weight children's academic achievement: a test of differential susceptibility versus diathesis stress. *J Child Psychol Psychiatry*, *56*(6), 693-701. https://doi.org/10.1111/jcpp.12331

Jaekel, J., Sorg, C., Breeman, L., Baumann, N., Bilgin, A., Bauml, J. G., & Wolke, D. (2021). Early regulatory problems and parenting: life-long risk, vulnerability or susceptibility for attention, internalizing and externalizing outcomes? *Eur Child Adolesc Psychiatry*, *30*(10), 1523-1531. https://doi.org/10.1007/s00787-020-01632-2

Jaekel, J., Wolke, D., & Chernova, J. (2012). Mother and child behaviour in very preterm and term dyads at 6 and 8 years. *Dev Med Child Neurol*, *54*(8), 716-723. https://doi.org/10.1111/j.1469-8749.2012.04323.x

Spanier, G. B. (1976). Measuring Dyadic Adjustment: New Scales for Assessing the Quality of Marriage and Similar Dyads. *Journal of Marriage and the Family*, *38*(1). https://doi.org/10.2307/350547

Wolke, D., Jaekel, J., Hall, J., & Baumann, N. (2013). Effects of sensitive parenting on the academic resilience of very preterm and very low birth weight adolescents. *J Adolesc Health*, *53*(5), 642-647. https://doi.org/10.1016/j.jadohealth.2013.06.014

© 2025 Wong, MKY et al. *JAMA Network Open*.

eAppendix. Inverse Probability Weighting

To adjust for potential selective dropout, the hierarchical multivariable Cox proportional hazards regression models were refitted using inverse probability weighting (IPW). Given that dropout was associated with low family SES in VP/VLBW adults and with male sex and low family SES in term-born adults (eTable 2), weights were generated via logistic regression that included family SES in VP/VLBW and sex and family SES in term-born control.

© 2025 Wong, MKY et al. *JAMA Network Open*.

eTable 1. Partnering Experiences of Full Sample for the 26- and 34-year Assessments

	**26-year Assessment (*n*=489)**			**34-year Assessment (*n*=416)**		
	**VP/VLBW (*n*=260)**	**Term-born (*n*=229)**	***P* value**	**VP/VLBW (*n*=214)**	**Term-born (*n*=202)**	***P* value**
Ever had sexual intercourse			<.001			<.001
Yes	191/257 (74.3%)	227/229 (99.1%)		168/206 (81.6%)	202/202 (100.0%)	
No	66/257 (25.7%)	2/229 (0.9%)		38/206 (18.4%)	0/202 (0.0%)	
Ever engaged in a serious romantic relationship			<.001			<.001
Yes	188/259 (72.6%)	221/229 (96.5%)		167/210 (79.5%)	199/202 (98.5%)	
No	71/259 (27.4%)	8/229 (3.5%)		43/210 (20.5%)	3/202 (1.5%)	
Being married/cohabiting			<.001			<.001
Yes	119/260 (45.8%)	148/229 (64.6%)		123/213 (57.7%)	173/202 (85.6%)	
No	141/260 (54.2%)	81/229 (35.4%)		90/213 (42.3%)	29/202 (14.4%)	

Notes. Data are presented as No./Total No. (percentage) of participants unless otherwise specified. Group differences were compared with chi-square tests.

Abbreviations: VP/VLBW, very preterm or very low birth weight.

© 2025 Wong, MKY et al. *JAMA Network Open*.

eFigure 1. Cumulative Incidence of First Partnering Experiences

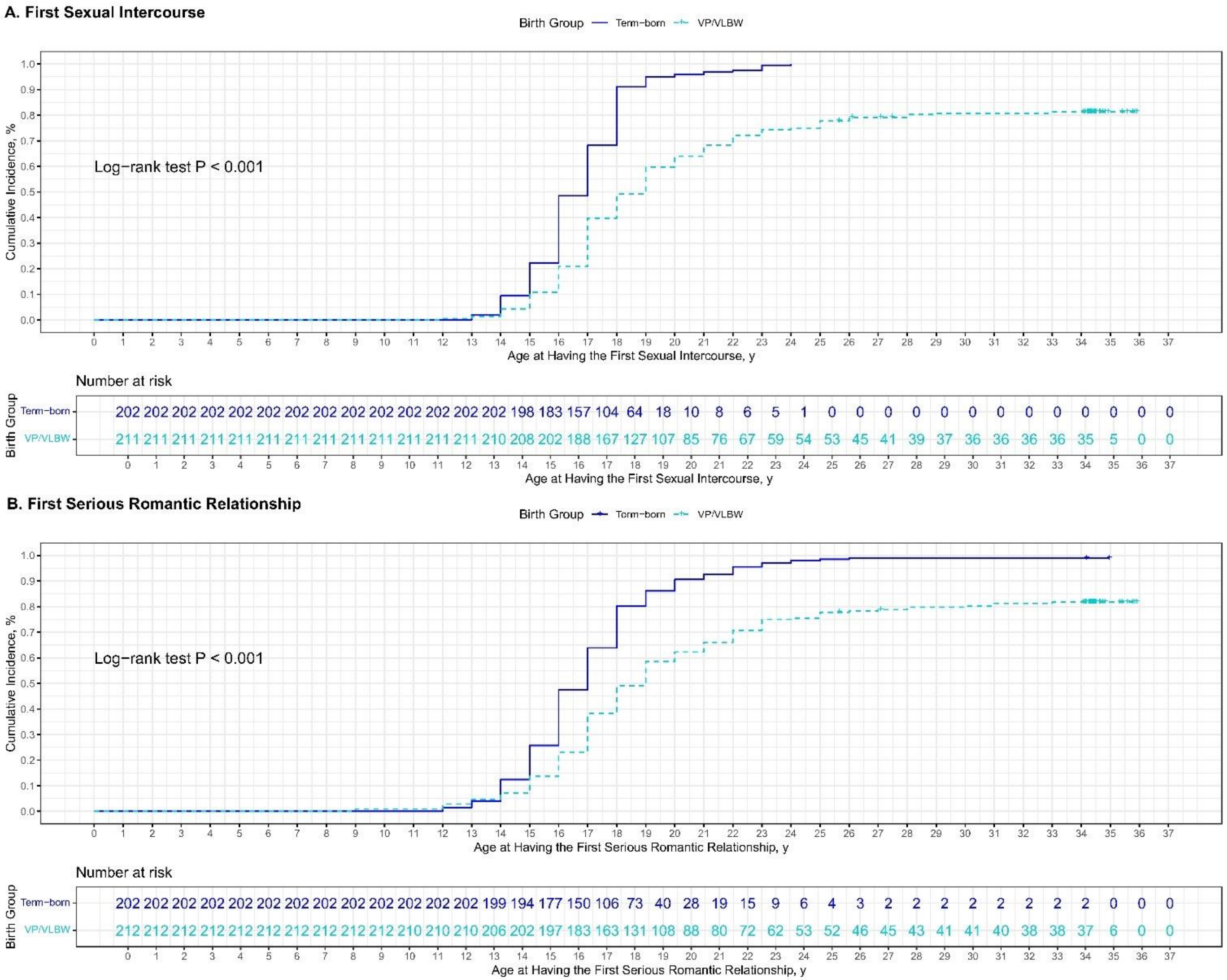

Figures show cumulative incidence (%) of having (A) the first sexual intercourse and (B) the first serious romantic relationship in VP/VLBW and term-born participants included in the analyses (*n*=414).

© 2025 Wong, MKY et al. *JAMA Network Open*.

eTable 2. Characteristics of Eligible Participants for the 34-year Assessment

	VP/VLBW (*n*=410)[a]			Term-born (*n*=306)[b]		
	**Participants** *n*=212 (51.7%)	**Dropouts** *n*=198 (48.3%)	***P* value**	**Participants** *n*=202 (66.0%)	**Dropouts** *n*=104 (34.0%)	***P* value**
Gestational age (weeks), mean (SD)	30.53 (2.06)	30.54 (2.52)	.98	39.64 (1.19)	39.64 (1.17)	>.99
Birth weight (grams), mean (SD)	1317.95 (311.43)	1283.18 (302.68)	.25	3360.69 (460.07)	3425.87 (419.93)	.23
Sex			.90			.004
Female	103/212 (48.6%)	95/198 (48.0%)		113/202 (55.9%)	40/104 (38.5%)	
Male	109/212 (51.4%)	103/198 (52.0%)		89/202 (44.1%)	64/104 (61.5%)	
Family SES			<.001			<.001
High	49/212 (23.1%)	33/198 (16.7%)		69/202 (34.2%)	22/104 (21.2%)	
Middle	103/212 (48.6%)	70/198 (35.4%)		87/202 (43.1%)	34/104 (32.7%)	
Low	60/212 (28.3%)	95/198 (48.0%)		46/202 (22.8%)	48/104 (46.2%)	
Presence of childhood NSI	43/209 (20.6%)	51/192 (26.6%)	.16	1/202 (0.5%)	0/104 (0.0%)	.47

Notes. Data are presented as No./Total No. (percentage) of participants unless otherwise specified. Independent sample t-test for continuous variables and chi-square test for categorical variables were performed.

Abbreviations: NSI, neurosensory impairment; SES, socioeconomic status; VP/VLBW, very preterm or very low birth weight.

[a] One VP/VLBW eligible adult participant died between 26- and 34-year assessments (see Figure 1).

[b] Two term-born eligible adult participants died between 26- and 34-year assessments (see Figure 1).

© 2025 Wong, MKY et al. *JAMA Network Open*.

eTable 3. Likelihood Ratio Tests of the Hierarchical Multivariable Cox Proportional Hazards Regression Models

**Overall Reproductive Window**	
**Step**	**Likelihood Ratio Tests**
1. Unadjusted estimate of VP/VLBW	χ^2 = 15.20, df = 1, *P* < .001
2. Adjusting for neonatal factors and childhood NSI	χ^2 = 7.23, df = 4, *P* < .001
3. Adjusting for childhood family factors	χ^2 = 0.76, df = 5, *P* = .58
4. Adjusting for sociodemographic factors	χ^2 = 15.86, df = 4, *P* < .001
**Early Reproductive Window (<30y)**	
**Step**	**Likelihood Ratio Tests**
1. Unadjusted estimate of VP/VLBW	χ^2 = 1.95, df = 1, *P* = .20
2. Adjusting for neonatal factors and childhood NSI	χ^2 = 7.61, df = 4, *P* < .001
3. Adjusting for childhood family factors	χ^2 = 0.86, df = 5, *P* = .51
4. Adjusting for sociodemographic factors	χ^2 = 8.32, df = 4, *P* < .001
**Late Reproductive Window (≥30y)**	
**Step**	**Likelihood Ratio Tests**
1. Unadjusted estimate of VP/VLBW	χ^2 = 15.66, df = 1, *P* < .001
2. Adjusting for neonatal factors and childhood NSI	χ^2 = 1.99, df = 4, *P* = .10
3. Adjusting for childhood family factors	χ^2 = 0.45, df = 5, *P* = .81
4. Adjusting for sociodemographic factors	χ^2 = 9.31, df = 4, *P* < .001

Notes. Neonatal factors included sex and family socioeconomic status. Childhood family factors included sibling status, cognitively stimulating parenting, maternal sensitivity, and parental partner relationship quality. Sociodemographic factors included educational level, relative poverty, and partnership status.

Abbreviations: NSI, neurosensory impairment; VP/VLBW, very preterm or very low birth weight.

© 2025 Wong, MKY et al. *JAMA Network Open*.

eTable 4. Univariable and Multivariable Analyses of Association of VP/VLBW and Individual Factors with Fertility During the Overall Reproductive Window

	Univariable Analysis		Multivariable Analysis	
	**HR (95% CI)**	***P* value**	**HR (95% CI)**	***P* value**
**VP/VLBW**	0.56 (0.42-0.76)	<.001	0.79 (0.57-1.09)	.15
**Neonatal Factors and Childhood NSI**				
Male sex	0.63 (0.47-0.84)	.002	0.66 (0.49-0.89)	.008
Family SES				
High (reference)	1	–	1	–
Middle	1.26 (0.89-1.79)	.20	1.26 (0.87-1.85)	.23
Low	1.57 (1.06-2.31)	.02	1.43 (0.94-2.17)	.10
Presence of childhood NSI	0.14 (0.05-0.38)	<.001	0.21 (0.08-0.59)	.003
**Childhood Family Factors**				
Any sibling				
No sibling (reference)	1	–	1	–
Had sibling since infancy	0.99 (0.64-1.53)	.96	1.04 (0.66-1.64)	.91
Had sibling since childhood	1.36 (0.87-2.13)	.18	1.34 (0.84-2.14)	.24
Low level of cognitively stimulating parenting	1.07 (0.73-1.58)	.71	0.95 (0.61-1.46)	.99
Low level of maternal sensitivity	0.94 (0.66-1.32)	.70	1.02 (0.71-1.47)	.94
Low level of parental partner relationship quality	1.04 (0.73-1.49)	.81	1.22 (0.83-1.78)	.22
**Sociodemographic Factors**				
Highest level of education achieved				
High (reference)	1	–	1	–
Medium	1.06 (0.77-1.46)	.74	1.21 (0.84-1.75)	.30
Low	0.49 (0.28-0.84)	.01	0.98 (0.55-1.76)	.90
Relative poverty	0.65 (0.46-0.90)	.01	0.88 (0.60-1.31)	.52
Married/cohabiting	5.68 (3.84-8.40)	<.001	4.86 (3.25-7.27)	<.001

Note. Participants with missing data were not included in the univariable analysis.

Abbreviations: CI, Confidence Interval; HR, Hazard Ratio; NSI, neurosensory impairment; SES, socioeconomic status; VP/VLBW, very preterm or very low birth weight.

© 2025 Wong, MKY et al. *JAMA Network Open*.

eTable 5. Percentage of Excess Risk Mediated by the Presence of Childhood NSI and Partnering in the Association of VP/VLBW with Lower Fertility during the Late Reproductive Window

Exposure	Mediator	HR (95% CI) confounder adjusted	HR (95% CI) confounder and mediator adjusted	PERM
VP/VLBW	Presence of childhood NSI	0.52 (0.34-0.80)	0.67 (0.44-1.03)	31.3%
VP/VLBW	Married/cohabiting	0.52 (0.34-0.80)	0.63 (0.41-0.97)	22.9%
VP/VLBW	Presence of childhood NSI, Married/cohabiting	0.52 (0.34-0.80)	0.78 (0.50-1.20)	54.2%

Notes. Percentage of excess risk mediated was calculated as $((HR_{\text{confounder adjusted}} - HR_{\text{confounder+mediator adjusted}})/(HR_{\text{confounder adjusted}} - 1)) \times 100\%$. Confounder adjusted models were adjusted for sex, family socioeconomic status, sibling status, cognitively stimulating parenting, maternal sensitivity, parental partner relationship quality, educational level, and relative poverty.

Abbreviations: CI, Confidence Interval; HR, Hazard Ratio; NSI, neurosensory impairment; PERM, percentage of excess risk mediated; VP/VLBW, very preterm or very low birth weight.

© 2025 Wong, MKY et al. *JAMA Network Open*.

eFigure 2. Sensitivity Analysis of Association of Fertility with VP/VLBW Estimated using Inverse Probability Weighting

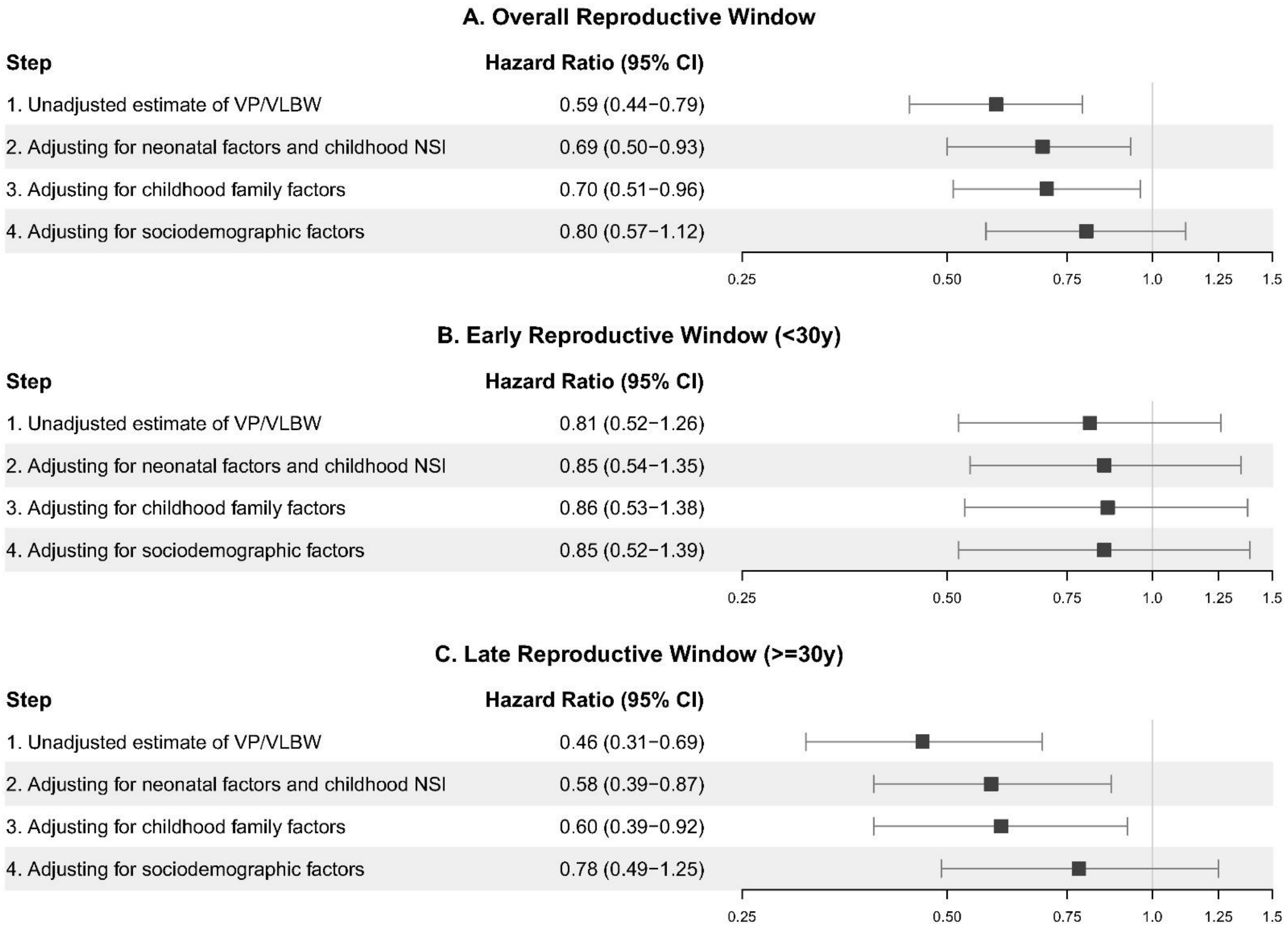

Figures show hazard ratios (HRs) and 95% confidence intervals (CIs) estimated using inverse probability weighting at each step of the hierarchical multivariable Cox proportional hazards regression models during the (A) overall, (B) early, and (C) late reproductive windows.

© 2025 Wong, MKY et al. *JAMA Network Open*.

eTable 6. Sensitivity Analysis of Association of VP/VLBW and Individual Factors with Fertility Estimated using Inverse Probability Weighting

	Overall Reproductive Window		Early Reproductive Window		Late Reproductive Window	
**Multivariable Analysis**	**HR (95% CI)**	***P* value**	**HR (95% CI)**	***P* value**	**HR (95% CI)**	***P* value**
**VP/VLBW**	0.80 (0.57-1.12)	.19	0.85 (0.52-1.39)	.52	0.78 (0.49-1.25)	.30
**Neonatal Factors and Childhood NSI**						
Male sex	0.68 (0.51-0.93)	.01	0.42 (0.26-0.69)	.001	1.00 (0.66-1.53)	.98
Family SES						
High (reference)	1	–	1	–	1	–
Middle	1.26 (0.86-1.84)	.24	1.80 (0.95-3.43)	.08	1.11 (0.68-1.83)	.67
Low	1.46 (0.95-2.23)	.08	2.13 (1.04-4.39)	.04	1.23 (0.70-2.16)	.48
Presence of childhood NSI	0.22 (0.07-0.68)	.009	0.52 (0.15-1.84)	.32	0.07 (0.01-0.54)	.01
**Childhood Family Factors**						
Any sibling						
No sibling (reference)	1	–	1	–	1	–
Had sibling since infancy	1.00 (0.62-1.62)	.99	0.67 (0.35-1.29)	.23	1.35 (0.69-2.68)	.39
Had sibling since childhood	1.28 (0.78-2.11)	.33	0.82 (0.42-1.61)	.56	1.82 (0.92-3.61)	.09
Low level of cognitively stimulating parenting	1.04 (0.68-1.58)	.86	1.14 (0.61-2.15)	.68	0.91 (0.51-1.62)	.74
Low level of maternal sensitivity	1.05 (0.71-1.55)	.81	0.88 (0.47-1.64)	.68	1.33 (0.80-2.21)	.28
Low level of parental partner relationship quality	1.27 (0.87-1.86)	.22	1.31 (0.72-2.36)	.38	1.16 (0.64-2.11)	.62
**Sociodemographic Factors**						
Highest level of education achieved						
High (reference)	1	–	1	–	1	–
Medium	1.20 (0.81-1.79)	.37	1.96 (1.03-3.72)	.04	0.79 (0.46-1.35)	.39
Low	0.83 (0.47-1.45)	.51	0.33 (0.08-1.30)	.12	1.27 (0.62-2.58)	.51
Relative poverty	0.98 (0.64-1.52)	.94	1.61 (0.84-3.09)	.16	0.64 (0.36-1.13)	.13
Married/cohabiting	5.19 (3.42-7.88)	<.001	6.67 (2.87-15.51)	<.001	4.68 (2.80-7.83)	<.001

Abbreviations: CI, Confidence Interval; HR, Hazard Ratio; NSI, neurosensory impairment; SES, socioeconomic status; VP/VLBW, very preterm or very low birth weight.

© 2025 Wong, MKY et al. *JAMA Network Open*.

eFigure 3. Subgroup Analysis of Association of Fertility with VP/VLBW Among Participants Who Were Partnered at 34-year Assessment

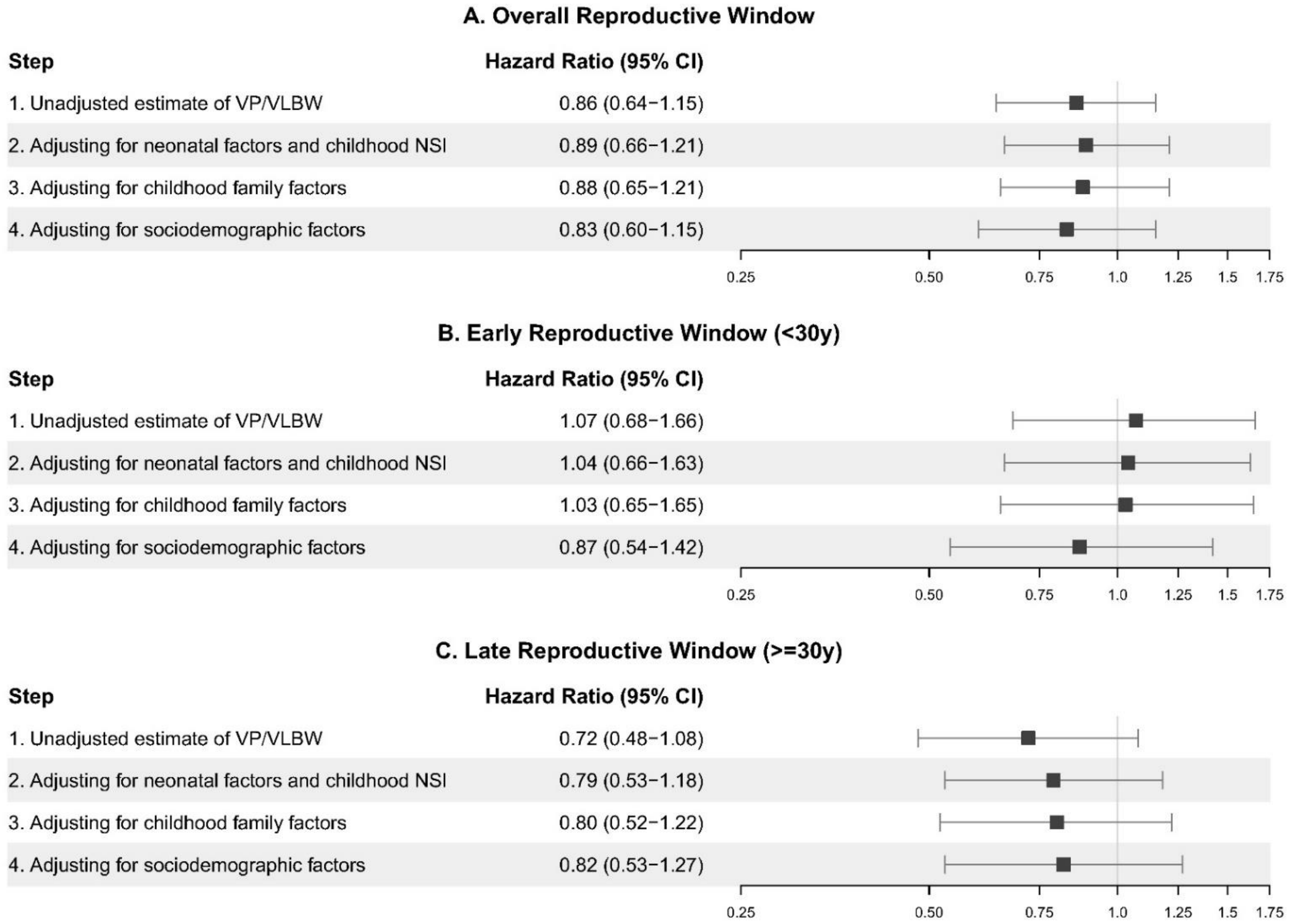

Figures show hazard ratios (HRs) and 95% confidence intervals (CIs) at each step of the hierarchical multivariable Cox proportional hazards regression models during the (A) overall, (B) early, and (C) late reproductive windows among the subgroup of participants who were partnered (i.e., married/cohabiting) at 34-year assessment (*n*=296).

© 2025 Wong, MKY et al. *JAMA Network Open*.

eTable 7. Subgroup Analysis of Association of VP/VLBW and Individual Factors with Fertility Among Participants Who Were Partnered at 34-year Assessment

	**Overall Reproductive Window**		**Early Reproductive Window**		**Late Reproductive Window**	
**Multivariable Analysis**	**HR (95% CI)**	***P* value**	**HR (95% CI)**	***P* value**	**HR (95% CI)**	***P* value**
**VP/VLBW**	0.83 (0.60-1.15)	.26	0.87 (0.54-1.42)	.59	0.82 (0.53-1.27)	.37
**Neonatal Factors and Childhood NSI**						
Male sex	0.72 (0.53-0.98)	.04	0.41 (0.25-0.67)	<.001	1.15 (0.76-1.75)	.51
Family SES						
High (reference)	1	–	1	–	1	–
Middle	1.32 (0.90-1.94)	.16	1.98 (1.01-3.89)	.05	1.10 (0.68-1.79)	.69
Low	1.40 (0.91-2.14)	.13	2.08 (1.00-4.33)	.06	1.14 (0.65-1.99)	.66
Presence of childhood NSI	0.35 (0.11-1.11)	.08	0.65 (0.16-2.74)	.56	0.17 (0.02-1.22)	.08
**Childhood Family Factors**						
Any sibling						
No sibling (reference)	1	–	1	–	1	–
Had sibling since infancy	0.91 (0.58-1.45)	.70	0.61 (0.32-1.18)	.15	1.25 (0.65-2.44)	.51
Had sibling since childhood	1.09 (0.68-1.75)	.73	0.73 (0.37-1.42)	.36	1.62 (0.81-3.22)	.18
Low level of cognitively stimulating parenting	1.31 (0.87-1.96)	.20	1.37 (0.74-2.51)	.32	1.17 (0.66-2.05)	.60
Low level of maternal sensitivity	0.86 (0.58-1.26)	.43	0.69 (0.39-1.22)	.21	1.19 (0.70-2.00)	.53
Low level of parental partner relationship quality	1.41 (0.96-2.09)	.08	1.67 (0.97-2.88)	.07	1.12 (0.65-1.95)	.68
**Sociodemographic Factors**						
Highest level of education achieved						
High (reference)	1	–	1	–	1	–
Medium	1.28 (0.89-1.84)	.19	2.06 (1.13-3.73)	.02	0.83 (0.51-1.34)	.44
Low	1.16 (0.63-2.13)	.63	0.55 (0.15-1.94)	.35	1.84 (0.87-3.90)	.12
Relative poverty	0.85 (0.57-1.27)	.42	1.26 (0.72-2.22)	.42	0.58 (0.32-1.04)	.07

Note. There were 296 out of 414 participants who were partnered (i.e., married or cohabiting) at 34-year assessment (see Table 1).

Abbreviations: CI, Confidence Interval; HR, Hazard Ratio; NSI, neurosensory impairment; SES, socioeconomic status; VP/VLBW, very preterm or very low birth weight.

© 2025 Wong, MKY et al. *JAMA Network Open*.
