# Supplementary material for: Fertility of Adults Born Very Preterm or With Very Low Birth Weight
Source: JAMA Netw Open. 2025 Mar 19;8(3):e251164. doi: 10.1001/jamanetworkopen.2025.1164 (PMC11923700; doi:10.1001/jamanetworkopen.2025.1164)
Supplement: Supplement 2. — Data Sharing Statement [file jamanetwopen-e251164-s002.pdf]

Data Sharing Statement

Wong. Fertility of Adults Born Very Preterm or With Very Low Birth Weight. *JAMA Netw Open*. Published March 19, 2025. doi:10.1001/jamanetworkopen.2025.1164

Data
Data available: Yes
Data types: Deidentified participant data, Data dictionary, Other (please specify)
Additional Information: On reasonable request to senior author de -identified data can be made available.
How to access data: D.Wolke@warwick.ac.uk
When available: With publication

Supporting Documents
Document types: None

Additional Information
Who can access the data: Researchers whose proposed use of the data has been approved
Types of analyses: Research
Mechanisms of data availability: Available via a signed data transfer agreement
